# Supplementary material for: Preoperatively Estimating the Malignant Potential of Mediastinal Lymph Nodes: A Pilot Study Toward Establishing a Robust Radiomics Model Based on Contrast-Enhanced CT Imaging
Source: Front Oncol. 2021 Jan 8;10:558428. doi: 10.3389/fonc.2020.558428 (PMC7821835; doi:10.3389/fonc.2020.558428)
Supplement: Supplementary file 1 [file DataSheet_1.pdf]

Supplementary Material

Contents

1. Supplementary A1: Eight nonzero radiomics features 2
   1.1 First order histogram feature: Percentile20 2
   1.2 Haralick feature: Sum Average 2
   1.3 Gray Level Cooccurrence Matrix (GLCM) features: GLCMEnergy, Cluster Shade, Inertia 2
   1.4 Gray Level Run Length Matrix (GLRLM) features: HighGrayLevelRunEmphasis (HGLRE), LongRunHighGrayLevelEmphasis (LRHGLE) 4
   1.5 Morphological feature: SurfaceArea 5
2. Supplementary A2: Feature selection 5
   2.1 Spearman correlation analysis with univariate logistic regression 6
   2.2 Spearman correlation analysis with GBDT and multivariate logistic regression 6
3. Supplementary A3: Radiomics model of non-enhanced images 7
4. Supplementary Figure 10
5. Supplementary Table 10

1. Supplementary A1: Eight nonzero radiomics features

1.1 First order histogram feature: Percentile20

X is a set of N_p voxels included in the region of interest (ROI).

N_p is the number of voxels in the image.

Percentile20 of X: the 20th percentile of X

1.2 Haralick feature: Sum Average

N_g is the number of discrete intensity values in the image.

i: a voxel of a given attenuation value; *j:* a voxel; *δ:* a distance of pixels; *θ:* angle

$$p_{x+y}(k) = \sum_{i=1}^{N_g} \sum_{j=1}^{N_g} p(i,j), \text{ where } i + j = k, \text{ and } k = 2,3,\dots,2N_g$$

P(i,j) is the cooccurrence matrix for an arbitrary δ and θ

p(i,j) is the normalized cooccurrence matrix and is equal to $\frac{\mathrm{P}(i,j)}{\sum \mathrm{P}(i,j)}$

sumAverage $= \sum_{k=2}^{2N_g} k p_{x+y}(k)$; sumAverage measures the relationship between the occurrences of pairs with lower intensity values and the occurrences of pairs with higher intensity values.

1.3 Gray Level Cooccurrence Matrix (GLCM) features: GLCMEnergy, Cluster Shade, Inertia

GLCM describes the number of times a voxel of a given attenuation value *i* is located

next to a voxel of j. A GLCM of size $N_g \times N_g$ describes the second-order joint probability function of an image region constrained by the mask and is defined as $P(i,j|\delta,\theta)$. The $(i,j)^{th}$ element of this matrix represents the number of times the combination of levels i and j occurs in two pixels in the image, which are separated by a distance of δ pixels along angle θ. The distance $\boldsymbol{\delta}$ from the center voxel is defined as the distance according to the infinity norm.

c is a value that shifts the intensities to prevent negative values in X.

X is a set of N_p voxels included in the ROI.

N_p is the number of voxels in the image.

$P(i,j)$ is the cooccurrence matrix for an arbitrary δ and θ.

$p(i,j)$ is the normalized cooccurrence matrix and is equal to $\frac{P(i,j)}{\sum P(i,j)}$.

Ng is the number of discrete intensity levels in the image.

$p_x(i) = \sum_{j=1}^{N_g} P(i,j)$ is the marginal row probability.

$p_y(j) = \sum_{i=1}^{N_g} P(i,j)$ is the marginal column probability.

μ_x is the mean gray level intensity of p_x and is defined as $\mu_x = \sum_{i=1}^{N_g} p_x(i)i$.

μ_y is the mean gray level intensity of p_y and is defined as $\mu_y = \sum_{j=1}^{N_g} p_y(j)j$.

$$p_{x+y}(k) = \sum_{i=1}^{N_g}\sum_{j=1}^{N_g} p(i,j), \text{ where } i+j=k, \text{ and } k = 2,3,\ldots,2N_g$$

g is a GLCM, where i,j are the spatial coordinates of g (i,j).

$\text{GLCMEnergy} = \sum_{i=1}^{N_p}(X(i) + c)^2$; GLCMEnergy is a measure of the magnitude of voxel values in an image. A larger value implies a greater sum of the squares of these values.

$\mathrm{ClusterShade} = \sum_{i=1}^{N_g} \sum_{j=1}^{N_g} \left(i + j - \mu_x - \mu_y\right)^3 \mathrm{p(i,j)}$; ClusterShade is a measure of the skewness and uniformity of the GLCM. A higher cluster shade implies greater asymmetry about the mean.

$\mathrm{Inertia} = \sum_{i,j}((i-j)^2 g(i,j))$; Inertia reflects the clarity of the image and texture groove depth. The contrast is proportional to the texture groove, and high values of the groove produce more clarity; in contrast, small values of the groove will result in low contrast and fuzzy images.

1.4 Gray Level Run Length Matrix (GLRLM) features: HighGrayLevelRunEmphasis (HGLRE), LongRunHighGrayLevelEmphasis (LRHGLE)

GLRLM describes gray level runs, which are defined as the length in number of pixels, of consecutive pixels that have the same gray level value. In a gray level run length matrix $P(i,j|\theta)$, the $(i,j)^{th}$ element describes the number of runs with gray level i and length j occurring in the image (ROI) along angle θ.

N_g is the number of discrete intensity values in the image.

N_r is the number of discrete run lengths in the image.

N_p is the number of voxels in the image.

$N_z(\theta)$ is the number of runs in the image along angle θ, which is equal to $\sum_{i=1}^{N_g} \sum_{j=1}^{N_r} \mathrm{P}(i,j|\theta)$and $1 \leq N_z(\theta) \leq N_p$.

$P(i,j|\theta)$ is the run length matrix for an arbitrary direction θ.

$p(i,j|\theta)$ is the normalized run length matrix, defined as $p(i,j|\theta) = \frac{\mathrm{P}(i,j|\theta)}{N_z(\theta)}$.

$\mathrm{HGLRE}(\theta) = \frac{\sum_{i=1}^{N_g} \sum_{j=1}^{N_r} \mathrm{P}(i,j|\theta) i^2}{N_z(\theta)}$; HighGrayLevelRunEmphasis (HGLRE) measures the distribution of the higher gray-level values, with a higher value indicating a greater concentration of high gray-level values in the image.

$\mathrm{LRHGLE}(\theta) = \frac{\sum_{i=1}^{N_g} \sum_{j=1}^{N_r} \mathrm{P}(i,j|\theta) i^2 j^2}{N_z(\theta)}$; LongRunHighGrayLevelEmphasis (LRHGLE) measures the joint distribution of long run lengths with higher gray-level values.

1.5 Morphological feature: SurfaceArea

$\mathrm{SurfaceArea} = \sum_{i=1}^{N} \frac{1}{2} |\mathrm{a}_i \mathrm{b}_i \times \mathrm{a}_i \mathrm{c}_i|$; SurfaceArea is an approximation of the surface of the region of interest (ROI) in mm^2, calculated using a marching cubes algorithm, where ***N*** is the number of triangles forming the surface mesh of the volume (ROI) and a_ib_i and a_ic_i are the edges of the ith triangle formed by points a_i, b_i and c_i.

2. Supplementary A2: Feature selection

We additionally used two other methods to select radiomics features. They were Spearman correlation analysis with univariate logistic regression, and Spearman correlation analysis with Gradient Boost Decision Tree (GBDT) and multivariate logistic regression.

2.1 Spearman correlation analysis with univariate logistic regression

After Spearman correlation analysis with univariate logistic regression, 14 radiomics features remained for modeling. The classification performances are as follows:

Supplementary Table S1. Spearman correlation analysis with univariate logistic regression

	Training Set	Test Set
Accuracy	0.786	0.803
f1_score	0.821	0.838
AUC	0.891	0.876
Sensitivity	0.821	0.861
Specificity	0.732	0.720
positive prediction	0.821	0.816
negative prediction	0.732	0.783

AUC, area under the receiver operating characteristic curve

2.2 Spearman correlation analysis with GBDT and multivariate logistic regression

After Spearman correlation analysis with GBDT and multivariate logistic regression, 10 radiomics features remained for modeling. The classification performances are as follows:

Supplementary Table S2. Spearman correlation analysis with GBDT and multivariate logistic regression

	Training Set	Test Set
Accuracy	0.857	0.803
f1_score	0.882	0.829
AUC	0.909	0.861
Sensitivity	0.893	0.806
Specificity	0.804	0.800
positive prediction	0.872	0.853
negative prediction	0.833	0.741

GBDT, Gradient Boost Decision Tree; AUC, area under the receiver operating characteristic curve.

3. Supplementary A3: Radiomics model of non-enhanced images

we used the same machine learning process as our main text to analyze the non-enhanced images. The progress included drawing ROI, features extraction, modeling and model evaluation. The classification performances are as follows:

Supplementary Table S3. Classification performances of radiomics model of non-enhanced image

	Training Set	Test Set
Accuracy	0.771	0.721
AUC	0.845	0.807
Sensitivity	0.821	0.667
Specificity	0.696	0.800

Note: AUC, area under the receiver operating characteristic curve

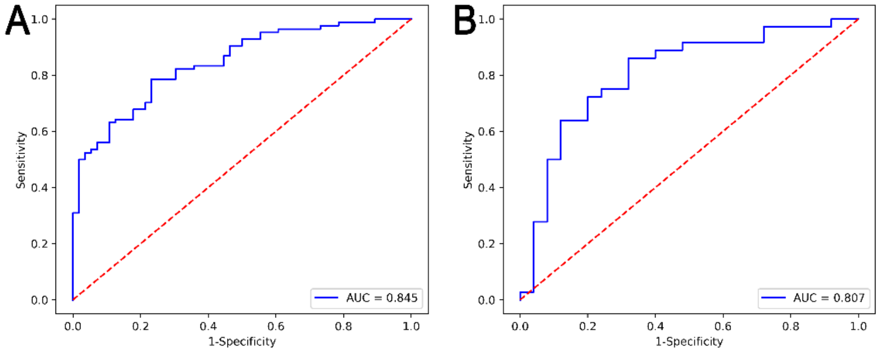

Supplementary Figure S1. Receiver operating characteristic curves for radiomics model of non-enhanced images. (A) The ROC curve of the training set. The AUC was 0.845. **(B)** The ROC curve of the test set. The AUC was 0.807. ROC = receiver operating characteristic, AUC = area under the ROC curve.

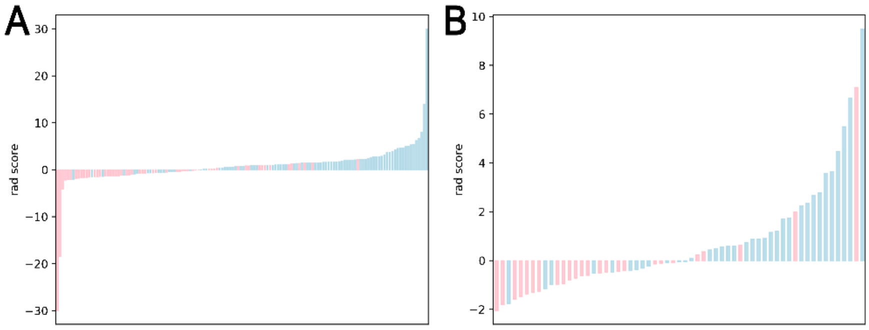

Supplementary Figure S2. Rad-score for lymph nodes. (A) Rad-score for every LN in the training set. **(B)** Rad-score for every LN in the test set. The y-axis refers to the Rad-score minus the optimal cutoff value. Up and down bars refer to the classified malignant and benign LNs, respectively. Blue and red bars refer to actual malignant and benign LNs, respectively. LN = lymph node.

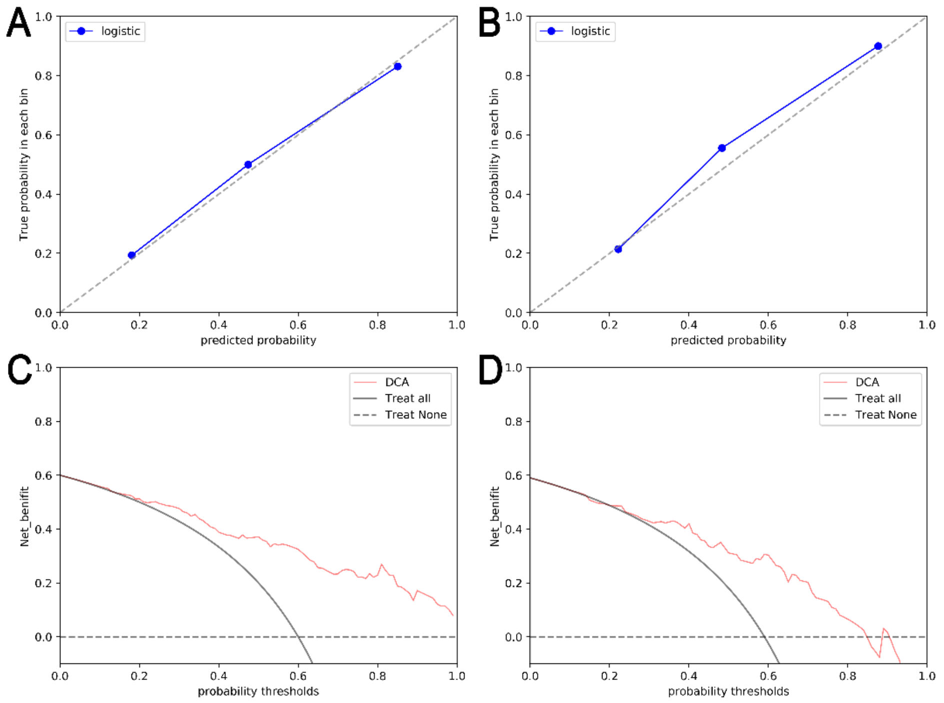

Supplementary Figure S3. Calibration curve and decision curve analysis of the radiomics model of non-enhanced images. (A) The calibration curve of the training set. **(B)** The calibration curve of the test set. The diagonal line represents a perfect prediction classification. **(C)** DCA of the training set. **(D)** The DCA of the test set. The y-axis indicates the net benefit. The net benefit is determined by calculating the difference between the expected benefit and the expected harm associated with each proposed model [net benefit = true positive rate – (false positive rate × weighting factor); weighting factor = threshold probability/(1-threshold probability)]. The solid gray line represents the assumption that all LNs are malignant. The gray dotted line represents the assumption that all LNs are benign. DCA = decision curve analysis, LN = lymph node.

4. Supplementary Figure

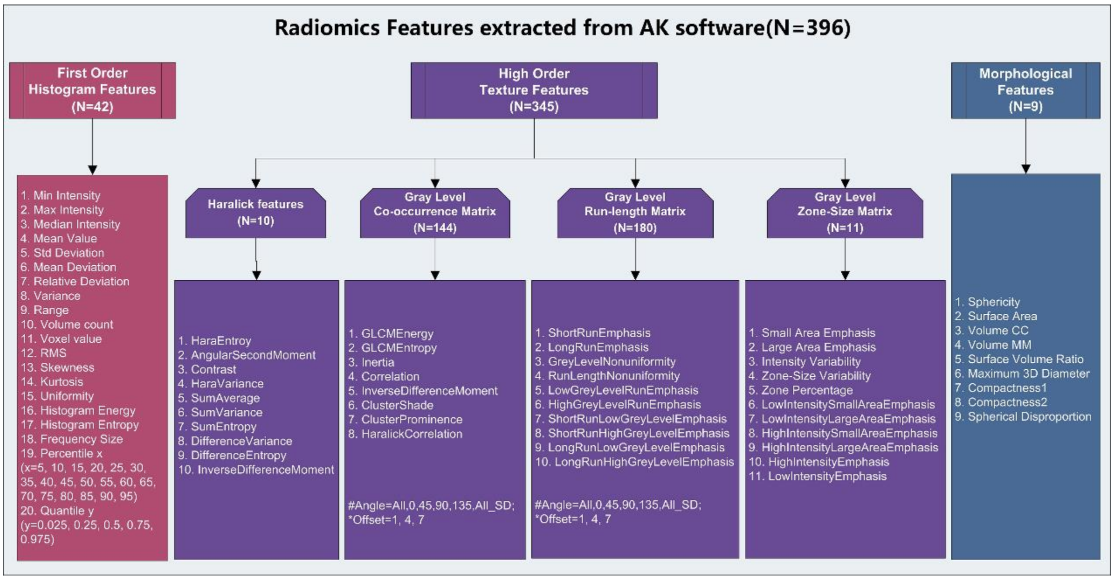

Supplementary Figure S4. Quantitative radiomics features in the study.

5. Supplementary Table

Supplementary Table S4. Comparison of the classification performances for different algorithms in the training set

	SVM	RF	LR	KNN	DT	NB
SVM	1	< 0.001	0.244	0.337	< 0.001	0.002
RF	< 0.001	1	< 0.001	< 0.001	< 0.001	< 0.001
LR	0.244	< 0.001	1	0.659	< 0.001	0.001
KNN	0.337	< 0.001	0.659	1	< 0.001	0.002
DT	< 0.001	< 0.001	< 0.001	< 0.001	1	0.107
NB	0.002	< 0.001	0.001	0.002	0.107	1

Note: SVM, support vector machine; RF, random forest; LR, logistic regression; KNN, k-nearest neighbors; DT, decision tree; NB, naive bayes.

Supplementary Table S5. Comparison of the classification performances for different algorithms in the test set

	SVM	RF	LR	KNN	DT	NB
SVM	1	0.023	0.960	0.031	0.003	0.060
RF	0.023	1	0.013	0.904	0.163	0.295
LR	0.960	0.013	1	0.039	0.001	0.064
KNN	0.031	0.904	0.039	1	0.399	0.435
DT	0.003	0.163	0.001	0.399	1	0.034
NB	0.060	0.295	0.064	0.435	0.034	1

Note: SVM, support vector machine; RF, random forest; LR, logistic regression; KNN, k-nearest neighbors; DT, decision tree; NB, naive bayes.
